# Acculturative stress and university life adjustment among international students in China: the moderating role of perceived social support

**DOI:** 10.3389/fpsyg.2025.1690645

**Published:** 2025-12-04

**Authors:** Hang Chen

**Affiliations:** Xi’an Eurasia University School of General Education, Xi’an, China

**Keywords:** acculturative stress, university life adjustment, social support, international students, China

## Abstract

This study examines how acculturative stress influences international students’ adjustment to university life in China, with a particular focus on the moderating role of perceived social support. Drawing on a sample of 642 international students, hierarchical regression and interaction analyses reveal that acculturative stress significantly impairs emotional, social, and academic adjustment. However, perceived social support buffers these effects, especially in emotional domains. These findings contribute to the literature on cross-cultural adaptation and underscore the importance of institutional and interpersonal support systems for student wellbeing.

## Introduction

With the rapid internationalization of higher education, China has become one of the top destinations for international students, attracting over 400,000 foreign learners before the COVID-19 pandemic. While many of these students bring strong academic motivation, they are often confronted with significant challenges in adjusting to the host country’s sociocultural and institutional environment. One of the most critical and frequently studied factors influencing their university adjustment is acculturative stress, which refers to the psychological strain experienced during cross-cultural transitions, including language difficulties, perceived discrimination, homesickness, and value conflicts (Su and Harrison, 2021; Ali et al., 2024; Jarrar and Nweke, 2025; Lerias et al., 2025).

Empirical research has consistently shown that acculturative stress is negatively associated with international students’ psychological wellbeing, academic engagement, and social integration (Tsegay et al., 2023). For instance, Su and Harrison (2021) found that Chinese students in the United States reported significantly lower quality of life and higher stress across four life domains when facing cultural dissonance. Similarly, Ali et al. (2024) reported that international students in Egypt experienced communication problems and academic disengagement as their cultural stress levels increased. Studies conducted in diverse national contexts, including Cyprus (Jarrar and Nweke, 2025) and Japan, further confirm the detrimental effects of acculturative stress on students’ ability to adjust to university life.

While the direct effects of acculturative stress have been well documented, growing attention has been given to protective and moderating factors that influence this relationship. Among these, perceived social support—defined as the subjective appraisal of emotional, informational, and instrumental assistance—has been widely recognized as a key resource in managing stress (Jarrar and Nweke, 2025; Han and Lee, 2023). In their study of Nigerian students in Cyprus, Jarrar and Nweke (2025) demonstrated that perceived social support not only reduced depressive symptoms but also moderated the relationship between stress and loneliness. Likewise, Han and Lee (2023) found that interpersonal support improved the psychological adjustment of Asian students in South Korea, emphasizing the importance of cultivating support networks in the host country.

Despite these findings, several gaps remain in the current literature. First, most studies have focused on international students in Western, English-speaking countries, such as the United States, the United Kingdom, and Australia (Su and Harrison, 2021; Ali et al., 2024). There is a noticeable lack of empirical studies on international students in China, where the sociocultural and institutional context is markedly different and may generate unique stressors for foreign students (Vora, 2023; Tsegay et al., 2023; Gao et al., 2025.

Second, although social support has been identified as beneficial, it has often been treated as an independent predictor or background factor. Only limited research has directly examined whether social support moderates the negative impact of acculturative stress on student adaptation (Jarrar and Nweke, 2025; Han and Lee, 2023). Third, much of the existing work treats “university adaptation” as a unidimensional construct. However, adaptation encompasses several distinct but interrelated domains, including academic adjustment, social functioning, and emotional wellbeing—each of which may respond differently to stress and support (Ali et al., 2024; Tsegay et al., 2023).

To address these gaps, this study investigates the moderating effect of perceived social support on the relationship between acculturative stress and university life adaptation among international students in China. It specifically examines whether students who report higher levels of social support experience less negative impact of acculturative stress on their adjustment to university life. Additionally, the study explores whether this moderation differs across three key subdomains of adaptation: academic, social, and emotional.

To address these gaps, this study investigates the moderating effect of perceived social support on the relationship between acculturative stress and university life adaptation among international students in China. It further examines whether this moderation differs across three domains of adaptation—academic, social, and emotional—thereby contributing to a more nuanced understanding of international students’ adjustment processes in a non-Western context.

## Foundation

### University life adaptation

The concept of university life adaptation encompasses how well students adjust to the academic, social, and emotional demands of higher education. One of the most widely used and empirically validated instruments to assess this construct is the Student Adaptation to College Questionnaire (SACQ), developed by Baker and Siryk (1984). This framework conceptualizes adjustment as a multidimensional process, reflecting students’ responses to various challenges encountered during their college experience.

The SACQ is structured around four key dimensions: academic adjustment, social adjustment, personal-emotional adjustment, and institutional attachment. Academic adjustment refers to a student’s ability to manage coursework, study effectively, and perform adequately in academic tasks. Social adjustment captures how well the student integrates into campus social life, including forming peer relationships and engaging in extracurricular activities. Personal-emotional adjustment focuses on the psychological and emotional wellbeing of the student, such as managing stress and coping with homesickness. Finally, institutional attachment reflects the student’s satisfaction with and commitment to their current institution (Baker and Siryk, 1984).

Empirical studies have applied the SACQ framework across diverse international populations. For instance, Tsegay et al. (2023) used these dimensions to assess international students’ adjustment experiences in non-Western educational settings, confirming the multidimensionality of adaptation. Similarly, Han and Lee (2023) demonstrated that social and emotional adjustment were more sensitive to contextual factors such as perceived discrimination and peer support than academic adjustment. These findings affirm the theoretical robustness of SACQ and its relevance to understanding the diverse ways in which students—particularly international students—navigate the transition to college life.

In this study, we adopt the SACQ conceptual model to operationalize university life adaptation. This allows for a comprehensive assessment of how acculturative stress and perceived social support influence different domains of adaptation. It also provides a theoretically grounded framework to evaluate whether specific types of adjustment (e.g., emotional or social) are more strongly affected by stress or more responsive to buffering mechanisms such as social support (Ra and Trusty, 2024; Jarrar and Nweke, 2025).

### Acculturative stress among international students

Acculturative stress refers to the psychological impact of adaptation to a new cultural environment, particularly among individuals navigating differences in language, norms, values, and social expectations. Koo (2021), which also examines acculturative stress in cultural adjustment contexts. For international students, this stress emerges as they attempt to reconcile the demands of the host culture with their cultural background, often leading to emotional and academic challenges. To systematically assess this construct, Sandhu and Asrabadi (1994) developed the Acculturative Stress Scale for International Students (ASSIS), a multidimensional instrument widely employed in cross-cultural research.

The ASSIS includes several dimensions of stress that are particularly salient for international students: perceived discrimination, homesickness, perceived hate or rejection, fear, culture shock, guilt, and difficulty in communicating. These components reflect both interpersonal and intrapersonal aspects of stress, encompassing the emotional burdens of isolation, cultural dissonance, and social exclusion (Sandhu and Asrabadi, 1994). Lim (2021), which provides additional evidence on acculturative stress factors.

Recent research has continued to affirm the relevance of these dimensions. For example, Çimsir (2024) found that international students experiencing higher levels of acculturative stress reported significantly more symptoms of depression, especially in relation to perceived discrimination and communication difficulties. Luz (2023), which highlights meaning-making processes within acculturative stress. Likewise, Ali et al. (2024) highlighted that acculturative stress predicts academic disengagement and emotional withdrawal, particularly in students with limited language proficiency or lack of cultural familiarity. Li and Liu (2021) further supported these findings by linking high acculturative stress to maladaptive coping strategies such as internet addiction.

Importantly, acculturative stress is not a uniform experience; its intensity and impact vary based on the student’s personal resources and environmental context. Studies such as Tsegay et al. (2023) have shown that the stress levels differ depending on the host country’s openness to diversity and the institutional support available to international students. These findings underscore the necessity of context-specific investigations, particularly in under-researched settings like China, where structural and cultural characteristics may uniquely shape international students’ adaptation experiences.

In this study, the ASSIS framework is adopted to measure the multifaceted nature of stress experienced by international students in China. This allows us to analyze how various stress dimensions interact with social support mechanisms and affect distinct aspects of university life adaptation.

### Perceived social support as a moderating factor

Perceived social support is widely recognized as a crucial protective factor in mitigating psychological distress and facilitating individual adaptation during transitional periods. Among the various models and measurement tools developed to understand social support, the Social Support Questionnaire for Transactions (SSQT) designed by Douglas et al. (1996) offers a multidimensional approach specifically tailored for understanding support in intercultural and high-stress contexts such as international education.

The SSQT conceptualizes social support as comprising several interrelated dimensions, including emotional support, informational support, instrumental support, and appraisal support. Emotional support reflects the availability of empathy and care from others; informational support refers to guidance or advice received; instrumental support captures tangible aid such as help with tasks; and appraisal support refers to constructive feedback or affirmation. These types of support are not only instrumental in reducing psychological distress, but also enhance individuals’ perceived capacity to cope with environmental demands (Douglas et al., 1996).

Multiple studies have substantiated the buffering role of social support in the context of acculturative stress. For instance, Ra and Trusty (2024) found that high levels of perceived social support significantly reduced the negative impact of acculturative stress on international students’ sense of belonging and academic motivation. Similarly, Jarrar and Nweke (2025) observed that social support moderated the effects of loneliness and stress on psychological wellbeing, acting as a stabilizing factor in emotionally challenging situations. Han and Lee (2023) further emphasized that support from peers and institutional services had a greater impact on emotional and social adjustment than on academic outcomes.

The buffering hypothesis of social support, originally articulated by Cohen and Wills (1985), posits that support is particularly beneficial when individuals face high levels of stress. This hypothesis is especially relevant in the case of international students, for whom support networks may be disrupted by geographic distance and cultural dislocation. The absence of culturally sensitive support services in host institutions can exacerbate students’ sense of isolation and cultural marginalization.

In this study, we adopt the SSQT framework to measure perceived social support and examine its moderating role between acculturative stress and university life adaptation. By doing so, we aim to test whether social support can alleviate the negative impact of stress across multiple dimensions of adjustment—academic, social, and emotional—among international students in China.

### Aims and hypothesis

Guided by the theoretical frameworks of the SACQ (Baker and Siryk, 1984), ASSIS (Sandhu and Asrabadi, 1994), and SSQT (Douglas et al., 1996), this study addresses the following research questions:

*RQ1*: How does acculturative stress influence international students’ adaptation to university life in China?

*RQ2*: Does perceived social support buffer the negative impact of acculturative stress on university life adaptation?

*RQ3*: Do these moderation effects vary across the academic, social, and emotional domains of adaptation?

Based on these research questions and prior literature, the following hypotheses were formulated:

*H1*: Acculturative stress is negatively associated with international students’ university life adaptation in China.

*H2*: Perceived social support moderates the relationship between acculturative stress and university life adaptation, such that the negative effect of stress is weaker for students with higher levels of support.

*H3*: The moderating effect of perceived social support varies across adaptation subdomains, with stronger effects on emotional and social adjustment than on academic adjustment.

By explicitly articulating these questions and hypotheses, the present study extends prior research by clarifying the stress–support mechanism underlying international students’ adaptation and by situating this process within the sociocultural context of Chinese higher education. By focusing on international students in China and examining the buffering role of perceived social support across multiple domains of adaptation, this study expands the existing literature on acculturation and student wellbeing. It also provides practical implications for student services and university policy aimed at fostering inclusive and supportive environments for globally mobile learners.

## Materials and methods

**FIGURE 1:**
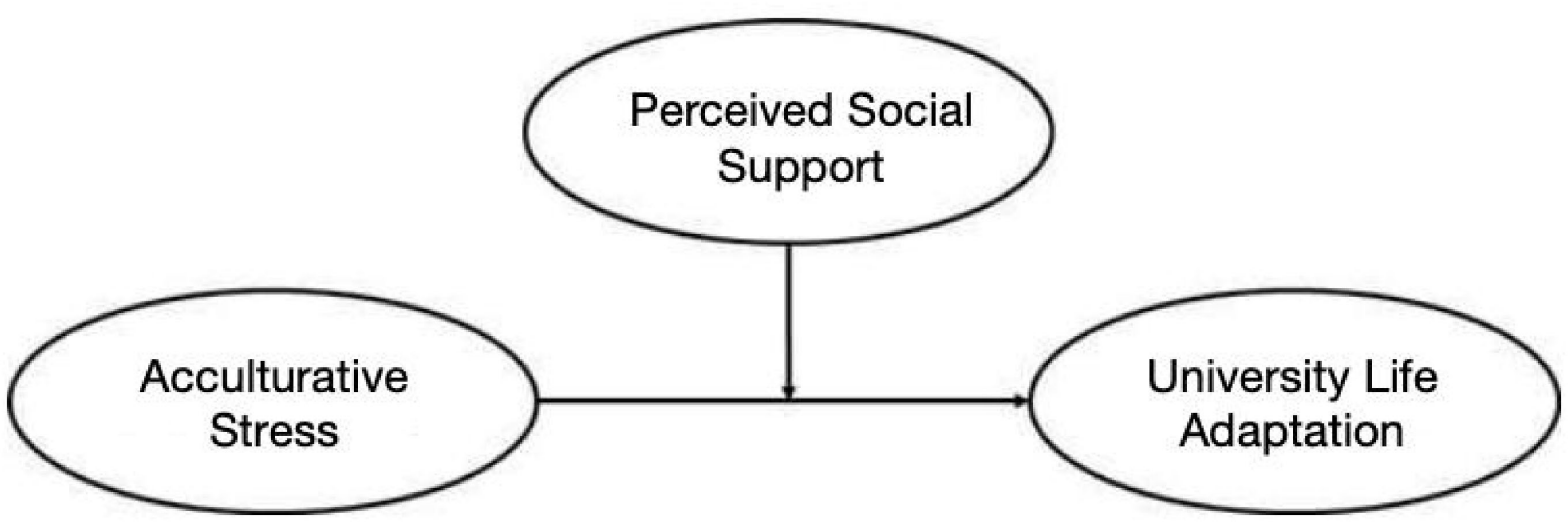
Conceptual model illustrating the hypothesized moderating role of perceived social support in the relationship between acculturative stress and university life adaptation.

This study aims to examine the impact of acculturative stress on international students’ university life adaptation in China, and to analyze the moderating role of perceived social support in this relationship. To achieve this, acculturative stress was defined as the independent variable, university life adaptation as the dependent variable, and perceived social support was set as the moderating variable. The conceptual model of the study is presented in Figure 1.

### Participants

This study surveyed 642 international students enrolled at 3, 4-year universities located in Region B of China. The universities were selected in cooperation with each institutionbetween national-student office, which facilitated on-site data collection. Paper-and-pencil questionnaires were distributed in person, and participation was voluntary and anonymous. All respondents were formally registered in their respective degree programs and possessed sufficient Mandarin proficiency to follow regular coursework. Table 1 summarizes the demographic characteristics of the study participants.

**TABLE 1:** Demographic characteristics of study participants.

Category	n	%
**Level of study**		
Undergraduate	520	81.0
Graduate (Master’s and Ph.D)	122	19.0
**Gender**		
Male	226	35.2
Female	416	64.8
**Year in program**		
Year 1	183	28.5
Year 2	96	15.0
Year 3	224	34.9
Year 4 or above	139	21.6
**Region of origin**		
South Korea	248	38.6
Russia and Central Asia	127	19.8
Southeast Asia	104	16.2
Africa	69	10.7
Europe	52	8.1
North and Latin America	42	6.5

### Instruments

University Life Adaptation was measured using the Student Adaptation to College Questionnaire (SACQ) developed by Baker and Siryk (1984), based on the Korean version translated by Hyun (1992). The original SACQ consists of 67 items covering four subdomains: Academic adjustment, social adjustment, personal-emotional adjustment, and institutional attachment. To improve participants’ understanding, the original 9-point scale was revised to a 5-point Likert scale ranging from 1 (“Not at all true”) to 5 (“Very true”).

In the present study, the primary analyses used the composite SACQ score to represent overall university life adaptation. In supplementary analyses, three key domains—academic, social, and emotional adjustment—were also examined separately to explore domain-specific moderation effects. The scale demonstrated excellent internal consistency, with a Cronbach’s α of 0.89 in the current study (compared to 0.94 reported by Yang, 2003).

Acculturative Stress was measured using the Acculturative Stress Scale for International Students (ASSIS) developed by Sandhu and Asrabadi (1994), adapted to the local context by Roh (2007). The scale consists of 33 items rated on a 5-point Likert scale (1 = “Strongly disagree,” 5 = “Strongly agree”). While the original scale includes seven subdomains—perceived discrimination, homesickness, perceived hate, fear, culture shock, guilt, and miscellaneous—this study used the total score without dividing into subscales. The Cronbach’s α for the scale was 0.96 in this study, compared to 0.92 reported by Roh (2007).

Perceived Social Support was assessed using the *Social Support Questionnaire for Transactions* (SSQT) developed by Douglas et al. (1996), based on the Korean-translated and revised version by the Iei (2016). This 18-item instrument uses a 5-point Likert scale ranging from 1 (“Strongly disagree”) to 5 (“Strongly agree”). It includes four dimensions: Emotional support for everyday issues (5 items), emotional support for problem situations (6 items), instrumental support for everyday issues (4 items), and instrumental support for problem situations (3 items). In this study, each type of support was assessed across three sources—Korean friends, co-national friends, and university professors—allowing for a multidimensional profile of perceived support. The overall internal consistency of the scale was 0.97 in this study. Subscale reliabilities were as follows: Korean friends (0.96), co-national friends (0.96), and professors (0.93), consistent with the IEI’s findings (overall α = 0.98).

To ensure the validity of the scales used in this study, an exploratory factor analysis (EFA) was conducted. For example, the EFA results for the overall social support scale indicated high sampling adequacy [Kaiser-Meyer-Olkin (KMO) = 0.918] and statistical significance of Bartlett’s test of sphericity (χ^2^ = 31,431.60, *p* < 0.001), confirming that the data were suitable for factor analysis. The extracted factors explained 75.92% of the total variance, and all items showed communality values above 0.46, suggesting that the items were well explained by the latent factors. Therefore, all items were retained for analysis. Additional EFAs were also conducted for each support source (Korean friends, co-national friends, professors) and each type of support (emotional vs. instrumental), and similar levels of construct validity were confirmed.

In the present study, adaptation was analyzed both as an overall composite score and, in supplementary analyses, by its three constituent domains—academic, social, and emotional adjustment. The main moderation model used the composite index to test the overall buffering effect of perceived social support, while domain-specific regressions examined whether moderation patterns differed across subscales.

### Procedure

This study was conducted through a structured questionnaire survey to examine the relationships among acculturative stress, perceived social support, and university life adaptation among international students in China. To accommodate participants’ linguistic diversity, the questionnaire was developed in three language versionscipants’ linguistic diversity, the questionnaire was developed in university life adaptation% completed the Chinese version, 22% the English version, and 14% the Korean version. To ensure comprehension, all participants had either passed the universityeveloped in university life *t*-test or submitted valid proficiency certificates (e.g., HSK Level 4 or above for Chinese-medium programs, IELTS ≥ 5.5 for English-medium programs).

A pilot test was conducted with 10 international students in China to evaluate the clarity, flow, comprehension, and cultural relevance of the questionnaire items. Based on their feedback, several minor wording adjustments were made with the assistance of bilingual graduate students fluent in both Korean and Chinese. The finalized version of the questionnaire was then distributed in paper format with the cooperation of international student affairs offices at participating universities. The survey took approximately 15–20 min to complete. Before participation, all respondents were informed of the studyrticipating universities. The survey edback, several as voluntary participants. The survey was administered only after participants provided informed consent. Prior to participation, all respondents were informed of the study’s purpose, content, data protection policy, and their rights as voluntary participants. The survey was administered only after participants gave informed consent.

### Data analysis

Although moderation was tested through hierarchical regression for transparency, these results were cross-validated using Hayes’ PROCESS macro (Model 1; Hayes, 2022). Both procedures yielded consistent results [interaction term *b* = 0.12, SE =0 .04, 95% CI (0.04, 0.20), *p* = 0.002), confirming the robustness of the moderation effect.

The collected data were analyzed using SPSS version 25.0. First, descriptive statistics and frequency analyses were conducted to examine the demographic characteristics of the participants. The internal consistency of each scale was assessed using Cronbach’s alpha coefficients. To investigate the relationships among the main variables, Pearson product-moment correlation analysis was conducted.

Prior to conducting regression analyses, the assumptions of normality, linearity, homoscedasticity, and independence were examined. Skewness and kurtosis values for all variables ranged between −1 and +1, indicating acceptable normality. Scatterplots of standardized residuals showed no serious violations of linearity or homoscedasticity, and the Durbin–Watson statistic (1.95) suggested independence of residuals. These checks confirmed that the data met the assumptions required for parametric analyses.

To test the effect of acculturative stress on university life adaptation and to examine the moderating role of perceived social support, Hayes (2022) PROCESS macro (Model 1) was utilized. Prior to moderation analysis, all continuous variables were mean-centered to minimize potential multicollinearity and to enhance the interpretability of interaction effects. To further ensure robustness, supplementary models were estimated controlling for demographic variables (gender, academic level, and region of origin) and length of stay in China. Inclusion of these covariates did not materially alter the direction or significance of the main effects or interactions, indicating the stability of the findings.

In addition to using the overall perceived social support score, moderation analyses were also conducted separately by support source—Korean friends, co-national friends, and university professors—to explore the distinct moderating effects of each type of support provider. The relative strength of moderation effects by support source was evaluated using the standardized regression coefficients (β) for the interaction terms and the change in explained variance (ΔR^2^), both of which are widely used indicators in moderation analysis (Baron and Kenny, 1986; Hayes, 2022).

Furthermore, to explore the moderating role of support quality in more detail, the four subdimensions of perceived support—emotional support for daily issues, emotional support for problem situations, instrumental support for daily issues, and instrumental support for problem situations—were also examined separately by source. This approach enabled a more nuanced analysis of the significance and size of the moderating effects across different types and providers of support.

To address potential construct overlap, we examined the discriminant validity of the ASSIS and SACQ scales. An exploratory factor analysis using principal axis factoring confirmed two distinct latent constructs, with all items loading above 0.60 on their respective factors and cross-loadings below 0.30. Variance inflation factors (VIFs) ranged from 1.87 to 2.42, well below the common threshold of 5, indicating that multicollinearity was not a critical concern. Additionally, the high negative correlations (*r* = –0.80∼–0.85) reflect the theoretically inverse relationship between acculturative stress and adjustment, rather than measurement redundancy.

## Results

### Descriptive statistics and correlations

Table 2 presents the descriptive statistics and Pearson correlation coefficients among the main study variables. The mean score of acculturative stress was 3.49 (*SD* = 0.67), indicating a moderate level of stress experienced by international students. Perceived social support had a mean of 3.56 (*SD* = 0.77), suggesting a relatively favorable perception of support. Among the three subdomains of university life adaptation, emotional adjustment had the highest mean (*M* = 3.63, *SD* = 0.82), followed by social adjustment (*M* = 3.28, *SD* = 0.80) and academic adjustment (*M* = 2.91, *SD* = 0.62).

**TABLE 2:** Descriptive statistics and Pearson correlations among study variables *(N* = 642).

Variable	M	SD	1	2	3	4	5	6
1. Acculturative stress	3.49	0.67	–					
2. Perceived social support	3.56	0.77	0.08*	–				
3. Emotional adjustment	3.63	0.82	–0.75**	0.49**	–			
4. Social adjustment	3.28	0.80	–0.80**	0.41**	0.86**	–		
5. Academic adjustment	2.91	0.62	–0.85**	0.15**	0.76**	0.76**	–	
6. Univ. life adaptation	3.28	0.60	–0.85**	0.39**	0.95**	0.95**	0.89**	–

M, Mean; SD, standard deviation. Univ. Life Adaptation refers to the mean of emotional, social, and academic adjustment scores. *p < 0.05; **p < 0.01 (two-tailed).

As expected, acculturative stress was significantly and negatively correlated with all adaptation outcomes: emotional adjustment (*r* = –0.75, *p* < 0.01), social adjustment (*r* = –0.80, *p* < 0.01), academic adjustment (*r* = –0.85, *p* < 0.01), and overall university life adaptation (*r* = –0.85, *p* < 0.01). These findings provide initial support for Hypothesis 1.

In contrast, perceived social support was positively associated with emotional (*r* = 0.49, *p* < 0.01), social (*r* = 0.41, *p* < 0.01), and academic adjustment (*r* = 0.15, *p* < 0.01), as well as with overall university life adaptation (*r* = 0.39, *p* < 0.01), indicating its potential buffering role. Additionally, the three adaptation subdomains were strongly interrelated (*r*s > 0.75), further supporting the validity of combining them into a composite index.

### Main effects regression

#### Moderation analysis

To test Hypothesis 2, a hierarchical multiple regression analysis was conducted to examine whether perceived social support moderates the relationship between acculturative stress and university life adaptation. All continuous variables were mean-centered prior to creating the interaction term to reduce multicollinearity. In Step 1, acculturative stress and perceived social support were entered as predictors. In Step 2, the interaction term between acculturative stress and perceived social support was added to the model.

As shown in Table 3, acculturative stress was a significant negative predictor of university life adaptation (β = –0.83, *p* <0.001), while perceived social support was a significant positive predictor (β = 0.24, *p* < 0.001). Importantly, the interaction term was also statistically significant (β = 0.12, *p* < 0.01), indicating that perceived social support moderated the relationship between acculturative stress and university life adaptation.

**TABLE 3:** Hierarchical multiple regression analysis predicting university life adaptation from acculturative stress, perceived social support, and their interaction.

Predictor	β (Step 1)	*p* (Step 1)	β (Step 2)	*p* (Step 2)
Acculturative stress (centered)	–0.92	< 0.001	–0.83	< 0.001
Perceived social support (centered)	0.42	<0.001	0.24	< 0.001
Interaction (stress × support)	–	–	0.12	< 0.01
Constant	3.27	< 0.001	3.27	< 0.001

#### Differential moderation across adaptation domains

To test Hypothesis 3, we conducted separate hierarchical regression analyses for each adaptation subdomain: emotional, social, and academic adjustment. Each model included centered acculturative stress, centered perceived social support, and their interaction term as predictors.

As shown in Table 4, the interaction between acculturative stress and perceived social support was statistically significant in the domains of emotional adjustment (β = 0.08, *p* < 0.001) and social adjustment (β = 0.07, *p* < 0.01), indicating that perceived social support buffered the negative effect of stress on both emotional and social adaptation. However, the interaction term was not significant in predicting academic adjustment (β = –0.04, *p* = 0.072). These results suggest that the protective role of social support is more pronounced in emotional and social domains of adjustment and less evident in the academic domain.

**TABLE 4:** Hierarchical regression analyses examining the moderating effect of perceived social support on the relationship between acculturative stress and three adaptation domains.

Adaptation domain	β (Stress)	*p* (Stress)	β (Support)	*p* (Support)	β (Interaction)	*p* (Interaction)
Emotional adjustment	–0.98	< 0.001	0.59	< 0.001	0.08	< 0.001
Social adjustment	–0.99	< 0.001	0.50	< 0.001	0.07	< 0.01
Academic adjustment	–0.79	< 0.001	0.17	< 0.001	–0.04	0.072

Taken together, these findings provide partial support for Hypothesis 3, highlighting the domain-specific nature of the moderating effect. In particular, perceived social support appears to mitigate the emotional and social strain associated with acculturative stress, whereas its influence on academic functioning may be more limited or indirect.

#### Simple slopes analysis

**FIGURE 2:**
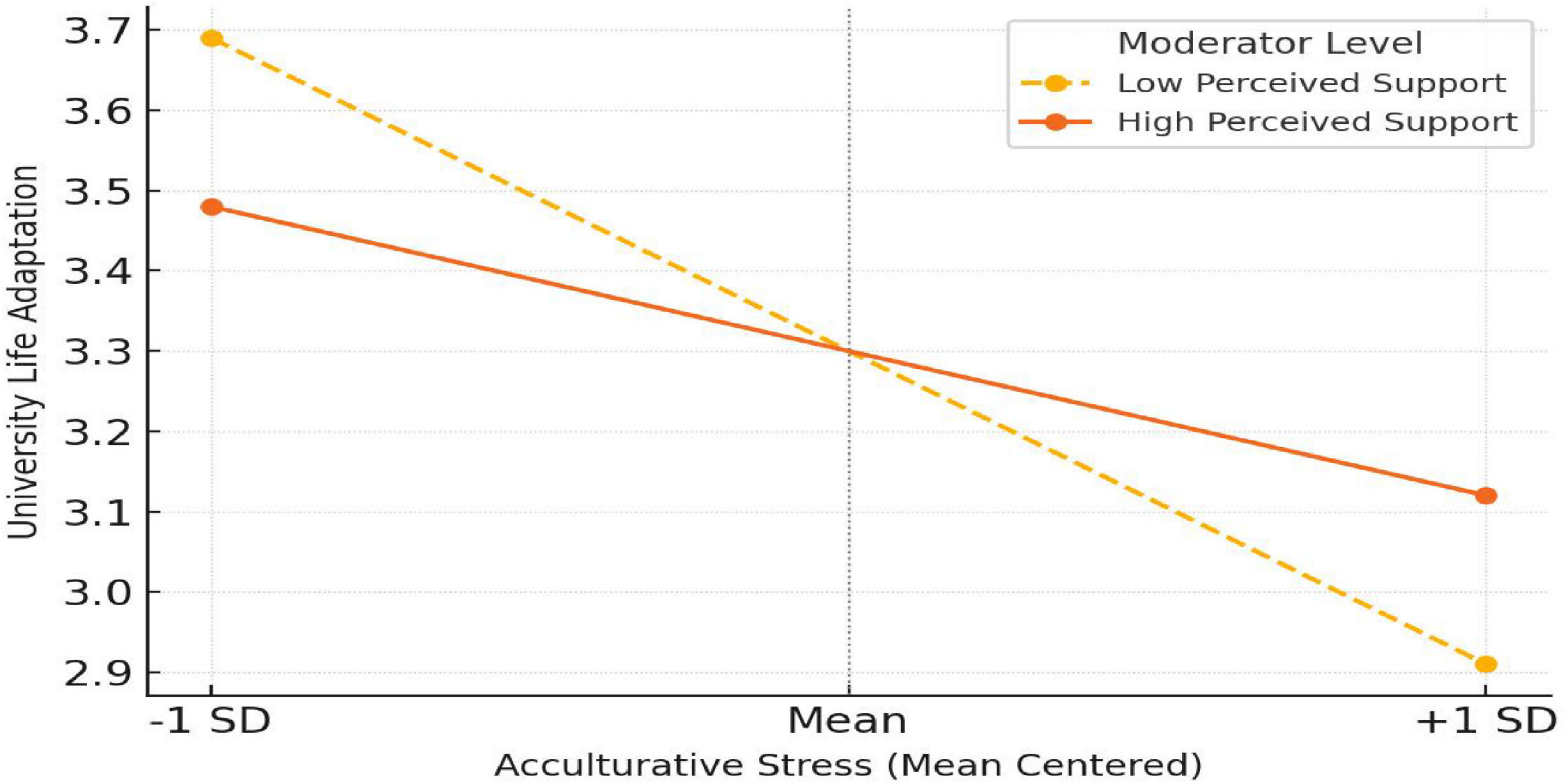
Interaction effect on university life adaptation.

To further interpret the interaction effect, a simple slopes analysis was conducted. As illustrated in Figure 2, the interaction between acculturative stress and perceived social support was significant, confirming H2. Specifically, acculturative stress was negatively associated with university life adaptation, but this relationship was moderated by perceived social support. Among students reporting low levels of social support, increases in acculturative stress were associated with a sharp decline in adaptation scores. In contrast, among those with high support, the negative association between stress and adaptation was attenuated, producing a flatter slope. This pattern supports the buffering hypothesis (Cohen and Wills, 1985), suggesting that perceived social support serves as a protective resource, mitigating the adverse impact of cross-cultural stress on international students’ overall adjustment.

**FIGURE 3:**
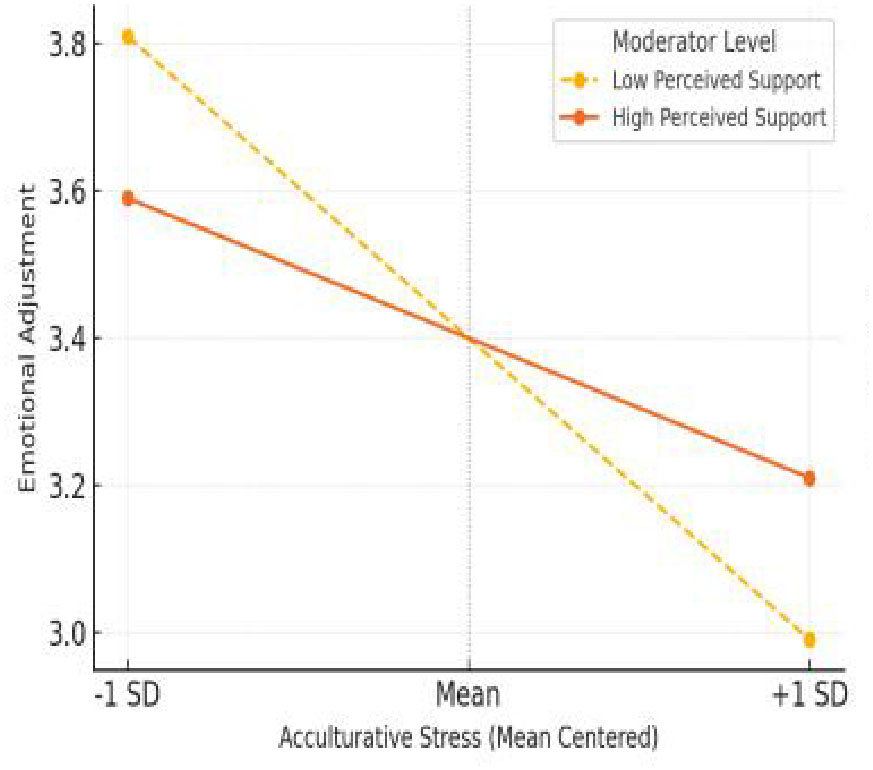
Interaction effect on emotional adjustment.

Figure 3 illustrates the interaction effect for emotional adjustment, where the moderation by perceived social support was most pronounced. For students perceiving low levels of support, emotional adjustment scores dropped sharply as acculturative stress increased. In contrast, students with high perceived support showed a much gentler decline, indicating better emotional regulation and resilience. This pattern demonstrates that social support is especially crucial for emotional wellbeing, consistent with previous studies showing its role in protecting against depression, anxiety, and psychological isolation (Ra and Trusty, 2024; Çimsir, 2024). These findings offer strong empirical support for H3, confirming that the buffering effect of support varies across adjustment domains and is particularly salient for emotional outcomes. Yang et al. (2023), which offers additional evidence on emotional conflict and regulation.

**FIGURE 4:**
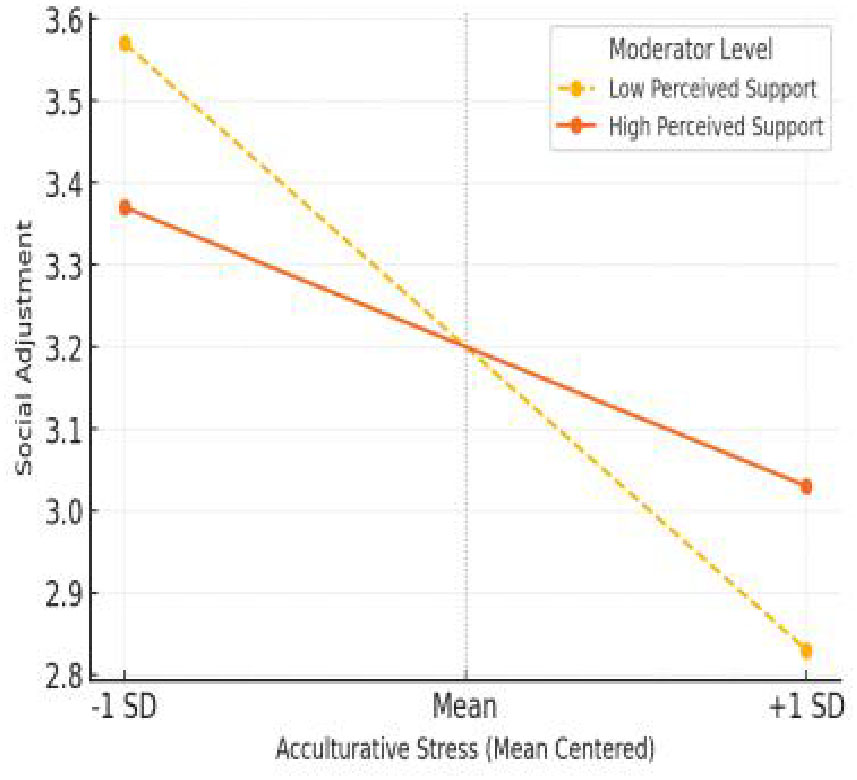
Interaction effect on social adjustment.

As shown in Figure 4, the interaction between acculturative stress and perceived social support was significant for social adjustment, offering strong support for H3. International students with low social support experienced a steeper decline in social adjustment scores as stress levels increased, whereas those with high support maintained relatively stable levels of social functioning. This finding highlights the importance of peer relationships, institutional community, and social inclusion in buffering the negative effects of cultural stress. It also aligns with prior research emphasizing that social domains are particularly responsive to interpersonal and contextual resources (Han and Lee, 2023; Jarrar and Nweke, 2025).

**FIGURE 5:**
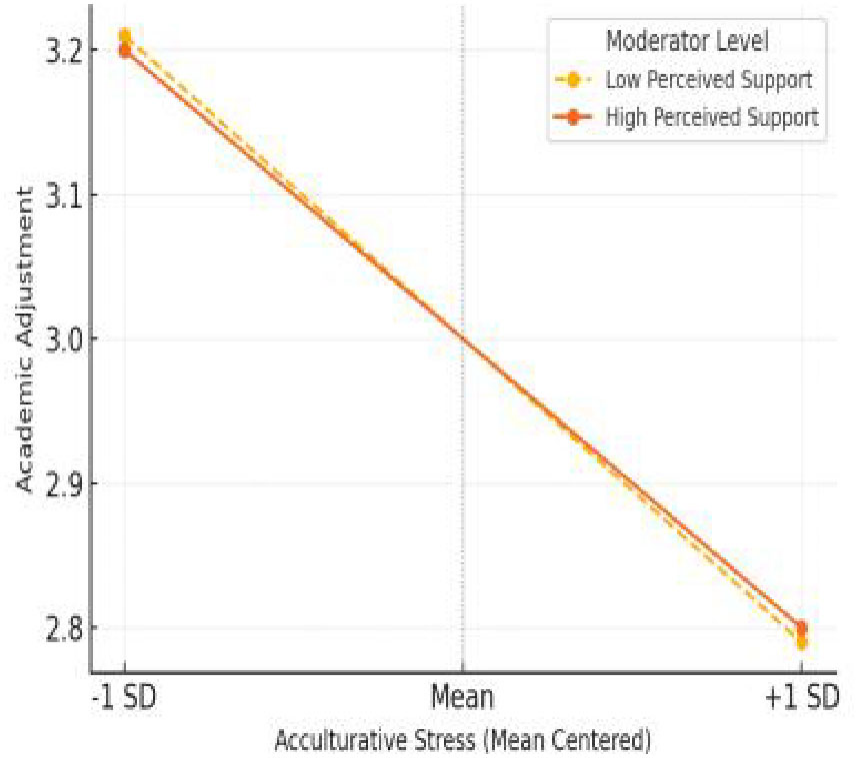
Interaction effect on academic adjustment.

Figure 5 presents the interaction between acculturative stress and perceived social support in predicting academic adjustment. The analysis revealed a non-significant interaction, indicating that perceived support did not substantially moderate the relationship between stress and academic adaptation. Both low and high support groups exhibited similar downward slopes as stress increased. This result suggests that, unlike emotional or social domains, academic adjustment may be less sensitive to variations in perceived support and may depend more on institutional factors, language proficiency, or academic preparedness. Thus, H3 is partially supported, as the moderation effect appears weak in the academic domain.

### Robustness checks/supplementary analysis

#### Robustness check: alternative operationalization of adaptation

To assess the robustness of the main findings, we re-estimated the moderation model using the three adaptation subdomains—emotional, social, and academic adjustment—as separate outcome variables rather than a composite score. The pattern of results was consistent with the primary analyses: the interaction between acculturative stress and perceived social support remained statistically significant for both emotional and social adjustment, but not for academic adjustment. This supports the claim that the buffering role of social support is domain-specific rather than uniform across all aspects of university life.

#### Supplementary analysis: gender as a covariate

To further ensure robustness, gender was included as a covariate in the moderation model. The inclusion of gender did not materially change the direction or significance of the results, indicating that the observed moderating effects were not confounded by gender differences.

## Discussion

This study investigated the relationship between acculturative stress and university life adaptation among international students in China, and examined the moderating role of perceived social support across three adaptation domains: academic, social, and emotional. Drawing on established theoretical frameworks such as the SACQ (Baker and Siryk, 1984), ASSIS (Sandhu and Asrabadi, 1994), and the SSQT (Douglas et al., 1996), the study addresses key gaps identified in prior research—particularly the lack of contextualized evidence from China, the neglect of multidimensional adaptation outcomes, and the limited exploration of moderation mechanisms. The findings supported Hypotheses 1 and 2 and partially supported Hypothesis 3, indicating that the moderating effect of perceived social support was domain-specific.

### Acculturative stress as a barrier to adaptation

Consistent with Hypothesis 1 and prior research (Su and Harrison, 2021; Ali et al., 2024; Jarrar and Nweke, 2025), the study found that acculturative stress is negatively associated with university life adaptation among international students in China. Students experiencing higher levels of stress—stemming from language difficulties, cultural dissonance, or perceived discrimination—were significantly less likely to adapt well to academic, social, and emotional challenges in the host environment. Yin and Ko (2023), which also discusses academic stress and psychological outcomes. These findings affirm the robustness of the acculturative stress framework across diverse cultural contexts and reinforce conclusions from studies conducted in Japan, Egypt (Ali et al., 2024), and Cyprus (Jarrar and Nweke, 2025).

Importantly, this study extends the literature by confirming that similar stress-related challenges persist in the Chinese higher education context, which remains under-researched. As Vora (2023) and Tsegay et al. (2023) have noted, structural barriers such as limited language access and rigid academic systems may intensify the adaptation burden for foreign students in China, making local investigations particularly urgent.

### The buffering role of perceived social support

In line with Hypothesis 2 and prior work grounded in the buffering hypothesis (Cohen and Wills, 1985; Han and Lee, 2023), this study found that perceived social support significantly moderated the negative impact of acculturative stress on overall university life adaptation. This finding supports previous studies showing that emotional and instrumental support from peers, professors, and institutional sources can reduce psychological strain and promote adjustment (Ra and Trusty, 2024; Jarrar and Nweke, 2025).

Notably, this study provides empirical validation for the SSQT framework (Douglas et al., 1996) in a Chinese context, indicating that international students who felt emotionally supported, socially included, or academically guided were more resilient to stressors commonly associated with cross-cultural transition. This affirms the need for host institutions to proactively cultivate support networks—not merely for logistical support but also for interpersonal and affective inclusion.

### Domain-specific moderation effects

The findings supported Hypotheses 1 and 2 and partially supported Hypothesis 3, indicating that the moderating effect of perceived social support was domain-specific—stronger in emotional and social adjustment than in academic adjustment. The findings supported Hypotheses 1 and 2 and partially supported Hypothesis 3, indicating that the moderating effect of perceived social support was domain-specific.

These findings align with previous research suggesting that emotional and social domains are more immediately responsive to interpersonal resources (Han and Lee, 2023), while academic adjustment is often shaped by structural factors such as language proficiency, prior learning experience, and pedagogical style (Ali et al., 2024; Tsegay et al., 2023). As such, academic difficulties may not be easily mitigated by peer or emotional support, highlighting the need for institutional interventions such as targeted academic mentoring, bilingual academic advising, or discipline-specific language support.

Furthermore, the differentiation between adaptation domains underscores the value of using the SACQ model (Baker and Siryk, 1984) to assess university life adaptation. Treating adaptation as a multidimensional construct—as advocated by Ra and Trusty (2024) and Tsegay et al. (2023)—enables more precise diagnoses and interventions.

Overall, these results partially confirm H3 and underscore the importance of differentiating between affective, social, and academic domains when examining the stress–support mechanism among international students.

### Contributions to theory and practice

Theoretically, this study contributes to a more differentiated and context-sensitive understanding of the stress–adaptation process. By demonstrating that the effects of acculturative stress are conditional on perceived support—and that these effects vary by domain—the findings advance integrative models of international student adjustment. This supports calls for ecological, culturally grounded models of acculturation (Çimsir, 2024; Li and Liu, 2021), particularly in under-examined contexts such as China.

Practically, the results suggest that universities must adopt multidimensional and targeted strategies to support international students. While emotional support (e.g., counseling, peer mentorship) is effective in reducing psychological strain, academic challenges may require institutional reforms in teaching, assessment, and advising. Social programs that facilitate intercultural friendships and inclusive campus cultures can also enhance students’ sense of belonging and mitigate the isolating effects of cultural stress.

Beyond statistical significance, the findings carry important practical relevance. Universities can enhance international studentslongiptation by developing structured peer−mentorship programs, bilingual counseling services, and cross-cultural community initiatives that foster emotional belonging and social integration. Training faculty advisors to provide culturally responsive guidance can further mitigate acculturative stress. Moreover, institutions may consider orientation workshops and intercultural communication courses that proactively strengthen perceived social support before challenges arise.

### Limitations and future directions

While this study offers valuable insights, several limitations warrant caution. First, the cross-sectional design precludes causal inference; longitudinal or experimental approaches are needed to establish temporal relationships among stress, support, and adaptation. Second, the convenience sample of students from three universities in one Chinese region limits generalizability to all international students in China or elsewhere. Future research should adopt more diverse and nationally representative sampling. Third, all variables relied on self-report measures, which may introduce social desirability or common-method bias. Combining self-report with behavioral or institutional data would strengthen validity.

Beyond these methodological limitations, the complexity of cultural adaptation may not be fully captured through quantitative approaches. Future studies should incorporate qualitative or mixed-methods designslimitaas interviews, focus groups, or case studieslto better understand studentsor case studiesltes and culturally shaped coping strategies. Comparative research across host countries would also help clarify contextual variations in the stress–support–adaptation process.

## Conclusion

This study examined how acculturative stress affects international students’ adaptation to university life in China and whether perceived social support moderates this relationship. The findings confirm that higher levels of acculturative stress hinder students’ academic, social, and emotional adjustment, while perceived social support buffers these negative effects—especially in the emotional and social domains.

By focusing on a non-Western context and adopting a multidimensional perspective, this study adds cultural specificity to existing acculturation models and highlights the importance of targeted support strategies. Universities should prioritize emotional and social support systems to enhance international students’ wellbeing, while also exploring structural solutions for academic integration.

## Data Availability

The raw data supporting the conclusions of this article will be made available by the authors, without undue reservation.
